# Supplementary material for: Genome-wide analyses of neonatal jaundice reveal a marked departure from adult bilirubin metabolism
Source: Nat Commun. 2024 Aug 30;15:7550. doi: 10.1038/s41467-024-51947-w (PMC11364559; doi:10.1038/s41467-024-51947-w)
Supplement: Supplementary file 3 — Description of Additional Supplementary Files [file 41467_2024_51947_MOESM3_ESM.pdf]

Description of Additional Supplementary Files

File Name: Supplementary Data 1
Description: Descriptive characteristics of the parent-offspring trios included in the study. Values are means (standard deviation) or count (proportion).

File Name: Supplementary Data 2
Description: Genome-wide significant variants associated with neonatal jaundice using the neonatal, maternal and paternal genomes in the Norwegian Mother, Father and Child Cohort study (using logistic regression). Neonatal jaundice was defined as receiving phototherapy treatment for jaundice. Replication was based on a meta-analysis of two Danish cohorts (Danish National Birth Cohort and Statens Serum Institut's genetic epidemiology). The haplotype-based analysis was based on parent-offspring data from the Norwegian Mother, Father and Child Cohort study. The lead SNP for the neonatal genome at chromosome two was not the one specified here, but this is a missense variant that has been selected among the lead SNPs. P-values are two-sided, and were not adjusted for multiple comparisons.

File Name: Supplementary Data 3
Description: Alternate allele frequency of a missense variant (rs6755571) associated with neonatal jaundice in different ancestries from the Norwegian Mother, Father, and Child Cohort study. Sample ancestry was inferred with a nearest centroid classifier (in the space of the first 3 PCs), trained on populations from the 1000 Genomes Project data. Neonatal samples (n = 28,112) are assigned to the nearest population by Euclidean distance to the centroid.

File Name: Supplementary Data 4
Description: Enrichment in evolutionary metrics identified using the GSEL pipeline. The Z-score indicates an enrichment (positive) or depletion (negative). The highest enrichment is found in the cross-population extended haplotype homozygosity scores. These scores, however, were not statistically significant. P-values are two-sided, and not corrected for multiple comparisons except specified (BH Corrected P-value column).

File Name: Supplementary Data 5
Description: Colocalization between neonatal jaundice and expression of 7 UGT1A* genes in 127 cell types/ tissues from the eQTL Catalogue. Posterior probabilities were obtained using coloc package. Sample size for neonatal jaundice was n = 27,384, cases = 1,826. eQTL dataset refers to the name of the data set, assay and tissue, extracted from the eQTL catalogue. Results are sorted by decreasing posterior probability of sharing the lead SNPs.

File Name: Supplementary Data 6
Description: Colocalization between neonatal jaundice and adult bilirubin levels and multiple phenotypes from the PAN UK Biobank at the UGT genes region. Posterior probabilities were obtained using coloc package. Sample size for neonatal jaundice was n = 27,384, cases = 1,826. Results are sorted by decreasing posterior probability of sharing the lead SNPs. Only

pairs of traits with a posterior probablity of association in the locus > 0.8 are shown (sum of posterior probability of distinct lead SNP and posterior probability of shared lead SNP).

File Name: Supplementary Data 7
Description: Definition of blood group according to two genetic variants. We used rs657152 instead of rs8176719, because the latter was not available in our data (indel), and the two variants are in strong LD, and in close proximity.

File Name: Supplementary Data 8
Description: Frequency of blood groups in mothers, fathers and neonates. Blood groups were determined genetically.
